# Sweet Taste Receptor Genetic Variation *TAS1R2* rs35874116 Is Associated with Dietary Quality in a Korean Population

**DOI:** 10.3390/nu18081224

**Published:** 2026-04-14

**Authors:** Eunyoung Kim, Jeong-Hwa Choi

**Affiliations:** Department of Food Science and Nutrition, Keimyung University, 1095 Dalgubeol-daero, Daegu 42601, Republic of Korea; eunyoung7026@naver.com

**Keywords:** diet quality, genetic variation, KHEI, sweet taste, *TAS1R2*

## Abstract

**Background/Objectives:** Individual differences in sweet taste sensitivity, influenced by genetic factors such as variants of the taste receptor type 1 member 2 (*TAS1R2*), are associated with food preferences and nutrient intake. However, the relationship between *TAS1R2* polymorphisms and diet quality in Koreans remains unexplored. This study investigated the association between the *TAS1R2* rs35874116 (T>C, Ile191Val) variant and diet quality, assessed using the Korean Healthy Eating Index (KHEI). **Methods:** Analyzing data from the Korean Genome and Epidemiology Study, we evaluated the dietary quality of 41,669 Koreans based on KHEI scores and *TAS1R2* rs35874116 genotypes (TT versus CT+CC). **Results:** The findings indicate that genetic variation in the sweet taste receptor is linked to specific components of dietary quality. Although total KHEI scores did not differ between genotypes, TT genotype carriers had significantly higher vegetable intake scores compared to C allele carriers (3.42 ± 1.35 vs. 3.37 ± 1.36, *p*_adjusted_ = 0.002). Additionally, TT carriers exhibited higher sodium intake (6.85 ± 3.53 vs. 6.95 ± 3.51, *p*_adjusted_ = 0.002) and lower scores in the moderation domain (18.82 ± 5.15 vs. 18.98 ± 5.07, *p*_adjusted_ = 0.002). **Conclusions:** The *TAS1R2* rs35874116 variant is associated with specific aspects of diet quality in Koreans, particularly vegetable and sodium intake. These findings suggest that genetic variations in sweet taste perception influence dietary behaviors among Koreans.

## 1. Introduction

A wide range of species, including humans, exhibit an innate preference for sweet-tasting foods and actively pursue their intake. Sweetness serves as a powerful stimulus, evoking positive sensory perceptions and pleasure, which significantly influence food choices and dietary behaviors [1]. Natural substances that elicit sweetness include glucose and certain amino acids; notably, sugars play a crucial physiological role as the primary energy source for the brain [2].

Humans perceive sweetness through receptors located in the oral cavity. The primary proteins involved in sweet taste signaling are part of the taste receptor type 1 (*TAS1R*, T1R) family, which is classified as G protein-coupled receptors (GPCR) [3]. This family includes three types, with taste receptor type 1 member 2 (*TAS1R2*, T1R2) and taste receptor type 1 member 3 (*TAS1R3*, T1R3) forming a heterodimer that mediates sweet taste recognition [3]. Key genetic variants associated with these receptors include rs35874116 (T>C, I191V) and rs9701796 (C>G, S9C) in *TAS1R2*, and rs307377 (C>T, C757A) in *TAS1R3*. Research indicates that these variants impact individuals’ food and nutrient intake [4,5,6]. Among these variants, rs35874116 in *TAS1R2* has been the focus of extensive research. Carriers of this variant demonstrate alterations in sweet taste sensitivity and preference, with trends differing based on obesity status [7]. Observational studies have further confirmed its link to food and nutrient consumption. For example, a longitudinal study involving Brazilian children found that those with the *TAS1R2* rs35874116 variant had distinct sugar intake patterns [4]. Similarly, research in a Mexican population showed significant differences in carbohydrate intake associated with the rs35874116 genotype [5]. An Italian survey indicated that obese individuals carrying the original C allele of rs35874116 had higher overall diet quality scores [7]. Additionally, the rs35874116 variant is linked to changes in the percentage of saturated fat in total energy intake, wine consumption, energy intake from sugars, and dietary fiber intake across various ethnic and national studies [7,8,9,10]. However, most studies have concentrated on specific nutrients and food items. Given that the *TAS1R2* variant influences not only sweet substances but also fat, dietary fiber, and overall nutrient intake, it could affect diet quality.

Emerging evidence indicates that assessing diet quality or patterns offers a clearer understanding of the relationship between diet and health than concentrating on individual nutrients or food intake [11]. Therefore, examining the link between *TAS1R2* genotypes and overall diet quality provides a more comprehensive view of how genetic variations affect eating behaviors. Despite this, only one relatively small-scale Italian study has explored the association between this genetic variant and diet quality [7], and no large-scale epidemiological studies have yet investigated the gene-diet relationship regarding overall diet quality. Building on our previous findings regarding the impact of bitter taste receptor type 2 member 38 (*TAS2R38*) variants on dietary habits in Koreans [12,13], this study investigates the relationship between the sweet taste receptor T1R2, also a member of the GPCR family, and dietary behaviors of the Korean population. Korean diets, known for their high intake of vegetables, grains, and sodium, differ significantly from Western diets [14]. However, genetic studies linking sweet taste receptors to diet quality in this population are scarce. To fill this research gap, we utilized data from the Korean Genome and Epidemiology Study (KoGES), a comprehensive nationwide cohort, to explore the genetic associations between *TAS1R2* variants and diet quality. The extensive nature of this representative cohort allows for a robust and statistically significant analysis of gene-diet relationships.

The primary aim of this study was to identify the association between the *TAS1R2* genetic variant rs35874116 and diet quality. We used the KoGES dataset to evaluate how this variant influences dietary patterns, employing the Korean Healthy Eating Index (KHEI) as an objective measure of overall diet quality.

## 2. Materials and Methods

### 2.1. Cohort Description

This study utilized data from the KoGES. KoGES is a nationwide cohort established by the Korea Disease Control and Prevention Agency (KDCA) to investigate gene-environment interactions in chronic diseases among Koreans [15]. We obtained epidemiological, dietary, and genetic information from various KoGES sub-cohorts, including the Ansan/Ansung study, Health Examinee study, and Cardiovascular Disease Association Study (No. CS00941-01).

**Figure 1:**
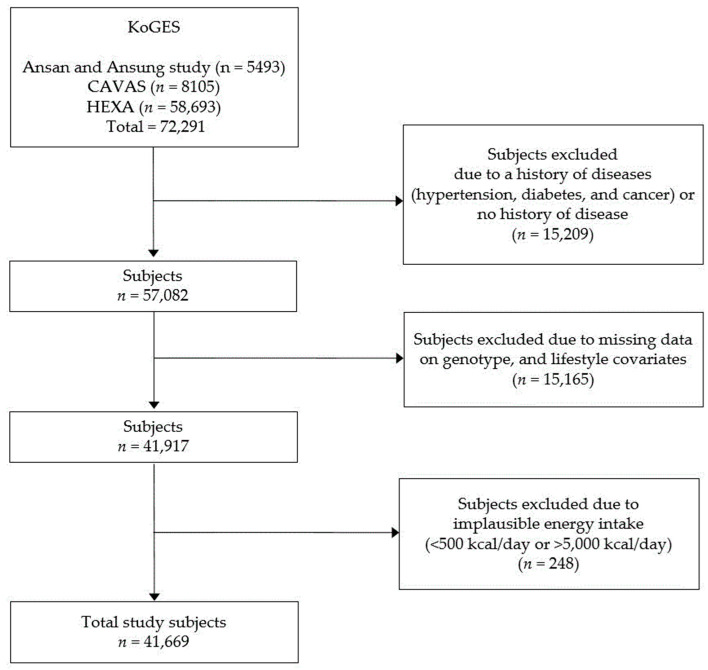
General characteristics of the study population according to *TAS1R2* rs35874116 genotype. CAVAS, Cardiovascular Disease Association Study; HEXA, Health Examinee study; KoGES, Korean Genome and Epidemiology Study; *n*, number of subjects.

Figure 1 illustrates the participant selection process for this study. Among the initial 72,291 participants with available genotype, epidemiological, and dietary data, the following exclusions were made: those with missing information on demographic and lifestyle factors (sex, age, body mass index (BMI), education level, cohabitation type, residence area, smoking and drinking status, and regular exercise) (*n* = 12,613); those with a medical history of hypertension, diabetes, or cancer (*n* = 15,141); those with no record of medical history (*n* = 68); those with missing physical activity information (*n* = 252); those with incomplete KHEI-related information (*n* = 2207); those with an energy intake of less than 500 kcal/d or more than 5000 kcal/d (*n* = 248); and those with missing *TAS1R2* rs35874116 genotype information (*n* = 93). Consequently, a total of 41,669 participants were included in the final analysis. This study was conducted following an exemption from the Institutional Review Board of Keimyung University (40525-202408-HR-049-02).

### 2.2. General Characteristics of Study Subjects

The information on the study subjects, provided by KoGES, included age, sex, BMI, education level, marital/cohabitation status, smoking and drinking behavior, and regular exercise. This data was reclassified and utilized for the analyses. To achieve statistical stability and address the low frequencies in certain response categories (e.g., marital status), specific variables were reclassified or merged as outlined earlier [13,16]. Briefly, education level was categorized as low for those with an elementary school education or less, middle for middle and high school graduates, and high for individuals with a university degree or higher. Marital and cohabitation status were classified as single for those who were single, separated, divorced, or widowed, and as married/cohabiting for those who were married or cohabiting with a partner. Lifestyle-related variables included smoking status, drinking status, and regular exercise. Smoking and drinking statuses were each classified into three categories: non-, ex-, and present. Regular exercise was defined as engaging in moderate or higher-intensity exercise for at least 30 min every day; otherwise, it was classified as no [15,17]. BMI was calculated by dividing body weight (kg) by the square of height (m^2^).

### 2.3. Genotyping and Genetic Data Analyses

This study obtained genotyping results analyzed by the KDCA. Participants were genotyped using the Korean Chip (K-chip 1.0) on the Korea Biobank Array platform (Affymetrix Axiom Array, Affymetrix, Santa Clara, CA, USA) [18]. The array consists of over 833,000 markers, including a dense collection of functional variants specific to Koreans. Standard quality control procedures were applied to the raw genotype data, resulting in the exclusion of individuals with sex inconsistencies, a sample call rate <95%, or excessive heterozygosity. Markers were also filtered out if their call rate <95% or if they significantly deviated from Hardy–Weinberg Equilibrium (HWE, *p* < 1.00 × 10^−6^). To improve genomic coverage, genotype imputation was performed using SHAPEIT v2 for phasing and IMPUTE v2 for the imputation analysis. The reference panel used was the 1000 Genomes Phase 3 data. Only imputed variants that met strict quality criteria—specifically, an imputation score (INFO < 0.8) and a minor allele frequency (MAF > 0.01)—were included in the final association analysis [18]. Data extraction and management were performed using PLINK (ver. 1.9) [19].

### 2.4. Dietary Data Collection and Korean Healthy Eating Index Analyses

Dietary data in the KoGES were collected using a validated food frequency questionnaire (FFQ) specifically designed for the study [20]. Participants reported their average consumption frequency across nine levels (ranging from almost never to three or more times per day) and portion sizes classified into three categories (small, standard, and large) for various food items over the past year. This intake data from the FFQ was converted into food and nutrient intakes based on the Dietary Reference Intakes for Koreans, utilizing the Korean Food Composition Table [21]. The processed data was then combined with other epidemiological and genetic information from the KoGES.

The KHEI is a tool developed to assess the overall diet quality of Korean adults and has become the most widely used index in Korea in recent years [22,23]. Created in 2015 based on the United States’ Healthy Eating Index, the KHEI underwent revisions in 2022 [24,25]. It evaluates 14 components across three domains: adequacy (8 items), moderation (3 items), and balance (3 items), with a maximum possible score of 100. Each component receives a maximum score (5 or 10 points) if the participant meets the intake levels or ratios specified by national guidelines. The adequacy domain (55 points) includes items such as breakfast frequency and the intake of mixed grains, fruits, and vegetables, with scores proportionally deducted for any shortfall in intake. The moderation domain (30 points) focuses on limiting sodium, saturated fatty acids, and sweets/beverages, with points deducted when intake exceeds recommended thresholds. The balance domain (15 points) assesses the percentage of energy derived from carbohydrates and fats, as well as total energy adequacy. For instance, if the energy ratio from carbohydrates falls outside the 55–65% range, points are deducted proportionally toward 0 as the ratio approaches 50% or 75%. Higher total KHEI scores indicate better adherence to dietary recommendations and superior overall diet quality.

### 2.5. Statistical Analyses

Participants’ general characteristics were compared by genotype using the Chi-squared test and the general linear model (GLM). To analyze the relationship between genotype and KHEI, we used an adjusted GLM with the *TAS1R2* genotype as a binary predictor (TT versus CT+CC) based on a dominant genetic model assumption. To improve the accuracy of our analysis and reduce potential confounding factors, we implemented a comprehensive statistical model that adjusted for various lifestyle and physiological variables, allowing us to identify the independent association between *TAS1R2* genetic variants and diet quality. The covariates included sex, age, education level, marital status, area of residence, alcohol consumption and smoking status, regular physical activity, BMI, and total energy intake. Before conducting the main analyses, we verified model assumptions, such as the normality of residuals and homoscedasticity, to ensure the reliability of our statistical estimates.

To reduce the risk of Type I errors due to multiple comparisons, we applied the Bonferroni correction. For the primary analysis of the 18 KHEI items, we set the significance level at *p* < 0.003 (0.003 ≈ 0.05/18). In the stratified analyses based on sex and obesity status, we adopted a more stringent threshold of *p* < 0.0014 (0.0014 ≈ 0.05/36, accounting for 18 KHEI items across two subgroups). Additionally, we tested interaction terms (genotype × sex and genotype × obesity status) within the GLM framework to assess potential effect modifications. Statistical analyses were conducted using SPSS software (Version 29.0, SPSS Inc., Chicago, IL, USA).

## 3. Results

### 3.1. General Characteristics of Study Subjects and Distribution of TAS1R2 Genotype

The distribution of the *TAS1R2* rs35874116 genotypes among the 41,669 participants was as follows: 30,790 had the TT genotype (73.9%), 10,035 had the CT genotype (24.1%), and 844 had the CC genotype (2.0%) (HWE *p* = 0.43). The MAF was 0.141, which aligns with findings from previous Korean studies [6,26]. The phenotypic effect of a genetic variant does not always exhibit a linear dose-response relationship, necessitating the consideration of various inheritance models to capture the underlying biological mechanisms [27]. In this study, the frequency of the homozygous risk genotype (CC) was only 2%, which made the application of recessive or additive models potentially unstable in terms of effect size estimates and statistical reliability. To address this, a dominant model (TT versus CT+CC) was adopted, ensuring a sufficient sample size in each analysis group and providing a more robust and stable evaluation of the variant allele’s impact within the population.

Table 1 presents the general characteristics of the participants based on the presence of the *TAS1R2* rs35874116 (T>C) C allele. The analysis indicated that lifestyle factors—such as sex, BMI, education level, cohabitation type, residence area, smoking and drinking status, and regular exercise—were independent of the rs35874116 genotypes (all *p* > 0.003).

### 3.2. Association Between TAS1R2 rs35874116 Genotypes and Diet Quality

Findings from the comparison of the *TAS1R2* rs35874116 genotypes revealed no meaningful difference in the total KHEI score between the groups. However, significant differences were identified in total vegetable intake, sodium intake, and the moderation domain, although the magnitude of these differences was relatively small (Table 2). Individuals with the *TAS1R2* rs35874116 TT genotype had a higher total vegetable intake compared to those with the CT+CC genotype, even after adjusting for general characteristics and lifestyle factors (3.42 ± 1.53 versus 3.37 ± 1.36, *p*_adjusted_ = 0.002). Furthermore, regarding sodium intake, the TT wild type, known to be more sensitive to sweetness [7,28], exhibited lower KHEI scores compared to the CT+CC genotype, indicating a tendency for higher sodium consumption (6.85 ± 3.53 versus 6.95 ± 3.51, *p*_adjusted_ = 0.002). In the moderation domain, which includes the sodium intake index, the CT+CC genotype also showed a significantly higher score than the TT genotype (18.82 ± 5.15 versus 18.98 ± 5.07, *p*_adjusted_ = 0.002).

Given previous reports indicating that the associations between genetic variants of taste receptors and diet or diseases differ based on sex and obesity status [7,12,13], we conducted further analyses stratified by these factors. In non-obese subjects, the CT+CC group showed a trend toward higher sodium intake scores and total moderation scores compared to the TT group; however, these associations did not reach statistical significance after applying the corrected *p*-level for stratified analyses. No significant results were found in the other stratified analyses (Supplementary Tables S1–S4). Lastly, there were no statistically significant interaction effects between *TAS1R2* genotype and sex, or between genotype and obesity status for any of the KHEI items (all *p* > 0.0014).

## 4. Discussion

This study investigated the relationship between the *TAS1R2* rs35874116 variant and diet quality among Koreans, using the KHEI as an objective assessment tool. Our findings indicate that this genetic variant in the sweet taste receptor is significantly associated with specific components of diet quality, extending beyond mere food preferences.

The human T1R2/T1R3 sweet taste receptor is a heterodimeric class C GPCR that detects a variety of stimuli, including natural sugars and artificial sweeteners [29]. Genetic variations in the *TAS1R* genes contribute to the functional diversity of these receptors by causing structural changes or affecting their expression levels in the plasma membrane. These alterations ultimately influence an individual’s sensitivity and physiological responses to sweetness [7,30]. Among these variants, rs35874116 (I191V) is situated within the N-terminal extracellular domain of T1R2 [31]. This amino acid change results in a partial loss of receptor function due to reduced receptor availability at the plasma membrane [29,32]. While this variant may not affect the receptor’s binding affinity for sweet stimuli [30], it has consistently been linked in observational studies to variations in food and nutrient intake, including sugar and carbohydrate consumption, as well as overall diet quality [4,5,7,29]. Additionally, this variant has also been associated with changes in physiological markers and disease risk, such as BMI [33], fasting insulin levels [33], and the risk of hypertriglyceridemia [5]. However, in the present study, when comparing diet quality scores of participants based on the *TAS1R2* rs35874116 genotype, no significant differences in total diet quality scores were observed. This contrasts with a previous study conducted in an Italian population, which found differences in total diet quality scores associated with the presence of the C allele [7]. However, the Italian study employed a diet quality index that emphasized the intake of fats and simple sugars and was performed with a relatively small sample size in a different culinary culture; these factors likely contributed to the differing outcomes between the two studies.

In this study, we identified significant differences in total vegetable intake, sodium intake, and the moderation domain score based on the presence of the *TAS1R2* rs35874116 C allele variant, although the actual score differences were relatively modest. Individuals with the TT genotype had a higher total vegetable intake compared to those with the C allele genotypes. This difference is likely linked to functional changes in sweet taste receptors, as described above. Prior research has indicated that the *TAS1R2* rs35874116 genetic variant affects sweet taste perception [4,7]. Specifically, the TT genotype is associated with a higher perceived intensity of sweetness, while carriers of the C allele are believed to have lower sensitivity to sweetness [7]. In our study, we found that individuals with the TT genotype, who are known to be more sensitive to sweetness, consumed more vegetables.

Previous reports on the relationship between sweet taste generic variation and the intake of bitter-tasting foods, such as vegetables, are limited. Most related studies have concentrated on *TAS2R38* bitter taste genetic variation to vegetables or compounds containing thiourea and their associations with bitter or sweet foods [34,35,36]. Nonetheless, a few studies have investigated the connection between sweet taste sensitivity and vegetable intake. The sweetness of sucrose can inhibit various bitter stimuli, an effect that is particularly evident when bitter compounds like caffeine are consumed alongside sweet substances [37,38,39]. Additionally, research has shown that girls sensitive to sweetness tend to have a higher preference for certain vegetables [40]. In a genotype-focused study, individuals with the *TAS1R3* rs35744813 CC wild-type genotype, who are more sensitive to sweetness, experienced a greater reduction in bitterness from sweetness compared to those with less sensitive genotypes [41]. Based on these findings, it is speculated that individuals with the TT genotype of the *TAS1R2* rs35874116 variant, who are known to be more sensitive to sweetness, may be better at detecting the natural sweetness in vegetables or sweetness from added seasonings. This heightened sensitivity could help mask the bitterness of vegetables, potentially increasing their preference and intake. Although we did not perform a sensory test for this genotype within this cohort, these insights suggest a connection between sweetness sensitivity and vegetable consumption.

The results of this study also indicated that individuals with the *TAS1R2* rs35874116 TT genotype tended to consume sodium more excessively than those with the CT+CC genotype. No prior research has established a connection between salt intake and the genetic variants related to sweet taste perception. However, the synergistic interaction of saltiness on sweetness is widely known [42]. A sodium-dependent glucose cotransporter was speculated to enhance sweet taste by increasing glucose uptake in the presence of sodium ions [43]. However, it has recently been reported that the human sweet taste perception mechanism is transmitted through T1R2/T1R3 dimers independently of sodium [44]. Although reports on the role of saltiness or sodium in the sweet taste perception mechanism are thus inconclusive, saltiness is known to bring about a synergistic effect on sweet taste intensity, and this connection between sweet and salty tastes has been confirmed in actual intake surveys in addition to the sensory tests. A study involving British children and adolescents found that having reduced salt intake (an average reduction of 3 g per day) resulted in a decrease in the consumption of sugar-sweetened beverages by more than two servings per week [45]. This suggests that a higher intake of salty flavors, such as sodium, may be linked to sensitivity to sweet tastes and the consumption of sweet foods. In this context, the increased sodium intake observed in individuals with the TT genotype can be understood within the framework of Korean dietary habits, particularly vegetable consumption. Data from the Korea National Health and Nutrition Examination Survey indicated that 24.5% of total sodium sources came from kimchi, a type of salted vegetable [46], making it the second-largest source of sodium [47]. Although there was no significant difference in kimchi consumption between the two genotype groups in this study, the TT genotype group had a slightly higher intake (146.55 ± 113.05) compared to the CT+CC group (143.60 ± 112.49). Although we did not perform the genotype-related sensory test, it is speculated that the high sweet taste sensitivity of the TT genotype enables individuals to perceive both the natural sweetness of vegetables and the flavors of seasonings, such as the sodium in kimchi. This perception may, in turn, influence their increased intake of both vegetables and sodium. Moreover, this trend likely impacted the moderation domain score, which includes a sodium intake evaluation item; the CT+CC genotype group scored significantly higher than the TT genotype group in this domain.

In this study, although the absolute differences in KHEI diet quality scores between *TAS1R2* genotypes were relatively small, clear variations were observed in the items regarding vegetable and sodium intake. Considering that the management of vegetable and sodium consumption is a critical dietary factor in the prevention and control of chronic diseases, these findings hold significant practical relevance. Our results suggest that individual genetic variations related to taste perception could be integrated into the planning and application of personalized nutrition management—precision nutrition—to enhance disease prevention strategies. However, to ensure the real-world applicability of these findings, further evidence must be accumulated through consistent results from diverse population-based studies.

In summary, the *TAS1R2* rs35874116 genetic variant is associated with the diet quality of Koreans, particularly in relation to vegetable intake, sodium consumption, and moderation in dietary choices. This study is, to our knowledge, the first large-scale epidemiological investigation to explore the relationship between *TAS1R2* sweet taste receptor variants and diet quality, rather than merely dietary intake. It utilizes data from approximately 41,000 participants in the KoGES cohort, providing significant explanatory power. The results contribute valuable evidence to our understanding of the genetic determinants of human dietary behavior. However, there are several limitations to consider when interpreting these findings. First, while we observed statistically significant differences, the actual variations in KHEI scores were relatively modest. This may be partly due to the high statistical power of our large study population. Nevertheless, this research is significant as it is the first to identify that *TAS1R2* genetic variants may influence specific dietary components, such as vegetable and sodium intake, and adherence to recommended levels, as assessed by the KHEI—a validated tool for evaluating diet quality in Koreans. These findings provide important primary data that support the idea that an individual’s taste genotype can affect dietary behavior. Second, because the dietary data were collected in the 2000s, current dietary habits among Koreans may have changed. Third, although the data were gathered using a validated FFQ, there may be inaccuracies and recall bias. Previous studies have indicated that FFQs have limitations in accurately estimating sodium intake [48]. Therefore, caution is warranted when interpreting the relationship between genotypes and sodium intake quality observed in this study. Fourth, this study was designed as a cross-sectional analysis. Given the established role of sweet taste receptors in dietary behavior and findings from prior literature, it can be inferred that genetic variations in these receptors may influence food intake and overall diet quality. However, the cross-sectional nature of the design makes it challenging to establish clear causal relationships. Fifth, the KoGES data only include Koreans aged 40 and older, which means the findings of this study do not adequately represent all age groups and ethnicities. Furthermore, although we adjusted for a wide range of lifestyle and environmental covariates and applied the Bonferroni correction to control for multiple comparisons in our statistical models to clarify the association between *TAS1R2* variants and diet quality, the possibility of Type I errors or residual confounding from unmeasured factors cannot be entirely ruled out. Lastly, as described earlier, due to the low MAF and the sparse distribution of the homozygous mutant genotype for *TAS1R2* rs35874116 in the Korean population, our main analyses were performed using a dominant model. Since the distribution of this genetic variant varies across ethnicities, further research employing alternative genetic models in diverse cohorts is necessary to gain a more comprehensive understanding of its influence on dietary behavior. Future research that incorporates a broader array of genetic and environmental factors is necessary to gain a more comprehensive understanding of how taste genetics may shape individual dietary behaviors. As such, caution is advised when interpreting the results of this study.

## 5. Conclusions

In this study, the *TAS1R2* rs35874116 genetic variant was associated with vegetable intake, sodium consumption, and the moderation domain. Our findings suggest that the *TAS1R2* rs35874116 variant plays a role in shaping specific components of dietary quality, such as vegetable and sodium intake, in addition to being related to sweetness-related behaviors. These results highlight the potential influence of taste genetics on diverse aspects of dietary behavior in the Korean population.

## Figures and Tables

**Table 1:** General characteristics of the study population according to *TAS1R2* rs35874116 genotype.

	Total (*n* = 41,669, 100%)	TT (*n* = 30,790, 73.9%)	CT+CC (*n* = 10,879, 26.1%)	*p*
Sex				
Male	14,091 (33.8)	10,392 (33.8)	3699 (34.0)	0.636
Female	27,578 (66.2)	20,398 (66.2)	7180 (66.0)	
Age	52.63 ± 8.12	52.63 ± 8.10	52.64 ± 8.19	0.870
BMI (kg/m^2^)	23.96 ± 2.90	23.95 ± 2.90	23.98 ± 2.91	0.472
Education				
Low	8289 (19.9)	6117 (19.9)	2172 (20.0)	0.841
Middle	21,121 (50.7)	15,610 (50.7)	5511 (50.7)	
High	12,259 (29.4)	9063 (29.4)	3196 (29.4)	
Cohabitation				
Single	963 (2.30)	698 (2.30)	265 (2.40)	0.309
Partner/married	40,706 (97.7)	30,092 (97.7)	10,614 (97.6)	
Residence				
Rural	5838 (14.0)	4320 (14.0)	1518 (14.0)	0.842
Urban	35,831 (86.0)	26,470 (86.0)	9361 (86.0)	
Smoking				0.906
Non-smoker	30,459 (73.1)	22,507 (73.1)	7952 (73.0)	
Ex-smoker	6001 (14.4)	4434 (14.4)	1567 (14.4)	
Present smoker	5209 (12.5)	3849 (12.5)	1360 (12.6)	
Alcohol drinking				
Non-drinker	21,148 (50.8)	15,611 (50.7)	5537 (50.9)	0.711
Ex-drinker	1470 (3.53)	1078 (3.50)	392 (3.60)	
Present drinker	19,051 (45.7)	14,101 (45.7)	4950 (45.5)	
Regular exercise				
No	20,251 (48.6)	14,927 (48.5)	5324 (48.9)	0.411
Yes	21,418 (51.4)	15,863 (51.5)	5555 (51.1)	

BMI, body mass index. Age and BMI are presented as mean ± standard deviation, while other variables are presented as number of subjects (%). *p* values were derived from general linear model for age and BMI, and from Chi-squared tests for all categorical variables.

**Table 2:** The KHEI scores of the study subjects according to *TAS1R2* rs35874116 genotype.

Domain/Items	Genotype (*n* = 41,669)		*B* (95%CI)	*p*_adjusted_
	TT (*n* = 30,790)	CT+CC (*n* = 10,879)		
Adequacy				
Having breakfast	8.07 ± 3.94	8.08 ± 3.94	−0.010 (−0.093, 0.073)	0.799
Mixed grains intake	2.76 ± 2.49	2.74 ± 2.49	0.025 (−0.028, 0.079)	0.350
Total fruit intake	2.97 ± 1.74	2.94 ± 1.74	0.022 (−0.012, 0.056)	0.212
Fresh fruit intake	3.03 ± 1.74	3.02 ± 1.74	0.017 (−0.017, 0.051)	0.332
Total vegetable intake	3.42 ± 1.35	3.37 ± 1.36	0.043 (0.016, 0.070)	0.002
Vegetable intake excluding kimchi and pickled vegetable intake	2.40 ± 1.35	2.37 ± 1.33	0.028 (0.002, 0.054)	0.030
Meat, fish, eggs, and beans intake	4.89 ± 2.66	4.86 ± 2.65	0.034 (−0.013, 0.081)	0.132
Milk and milk product intake	5.46 ± 4.06	5.50 ± 4.05	−0.038 (−0.122, 0.045)	0.399
Total scores of the adequacy	33.01 ± 10.14	32.89 ± 10.12	0.122 (−0.015, 0.295)	0.159
Moderation				
Ratio of white meat to red meat	2.76 ± 2.76	2.76 ± 2.77	−0.002 (−0.062, 0.057)	0.954
Sodium intake	6.85 ± 3.53	6.95 ± 3.51	−0.107 (−0.174, −0.041)	0.002
Percentage of energy from sweets and beverages	9.21 ± 2.48	9.26 ± 2.39	−0.047 (−0.095, 0.001)	0.053
Total scores of the moderation	18.82 ± 5.15	18.98 ± 5.07	−0.157 (−0.255, −0.059)	0.002
Energy balance				
Percentage of energy from carbohydrates	2.01 ± 1.99	1.98 ± 1.98	0.027 (−0.014, 0.069)	0.173
Percentage of energy intake from fat	2.87 ± 2.15	2.86 ± 2.15	0.011 (−0.033, 0.055)	0.591
Energy intake	3.63 ± 2.01	3.63 ± 2.02	0.002 (−0.042, 0.046)	0.940
Total scores of the balance	8.51 ± 4.42	8.48 ± 4.41	0.040 (−0.050, 0.131)	0.924
Total scores of KHEI	60.34 ± 12.27	60.34 ± 12.25	0.005 (−0.221, 0.232)	0.924

*B*, unstandardized coefficient; CI, confidence interval; KHEI, Korean Healthy Eating Index. Values are presented as mean ± standard deviation. *p*_adjusted_ values are from adjusted general linear model controlling for covariates including sex, age, education, cohabitation, residence, alcohol drinking, smoking, regular exercise, body mass index and total energy intake.

## Data Availability

This study analyzed the data of KoGES from KDCA. Requests to obtain the datasets should be made to the KDCA (https://coda.nih.go.kr/frt/index.do, accessed on 25 February 2026).

## Supplementary Materials

The following supporting information can be downloaded at: https://www.mdpi.com/article/10.3390/nu18081224/s1, Table S1: KHEI scores of male study subjects by *TAS1R2* rs35874116 genotype; Table S2: KHEI scores of female study subjects by *TAS1R2* rs35874116 genotype; Table S3: KHEI scores of non-obese subjects by *TAS1R2* rs35874116 genotype; Table S4: KHEI scores of obese subjects by *TAS1R2* rs35874116 genotype.

## Abbreviations

The following abbreviations are used in this manuscript:

*B*	Unstandardized coefficient
BMI	Body mass index
CAVAS	Cardiovascular Disease Association Study
CI	Confidence interval
FFQ	Food frequency questionnaire
GLM	General linear model
GPCR	G protein-coupled receptors
HEXA	Health Examinee study
HWE	Hardy–Weinberg Equilibrium
KDCA	Korea Disease Control and Prevention Agency
KHEI	Korean Healthy Eating Index
KoGES	Korean Genome and Epidemiology Study
*TAS1R*, T1R	Taste receptor type 1 family
*TAS1R2*, T1R2	Taste receptor type 1 member 2
*TAS1R3*, T1R3	Taste receptor type 1 member 3
*TAS2R38*	Bitter taste receptor type 2 member 38
