# Supplementary material for: Sweet Taste Receptor Genetic Variation TAS1R2 rs35874116 Is Associated with Dietary Quality in a Korean Population
Source: Nutrients. 2026 Apr 14;18(8):1224. doi: 10.3390/nu18081224 (PMC13118574; doi:10.3390/nu18081224)
Supplement: Supplementary file 1 [file nutrients-18-01224-s001.zip › nutrients-4200659-supplementary.pdf]

Supplementary Table S1. KHEI scores of male study subjects by *TAS1R2* rs35874116 genotype

Domains	Items	Males(n=14,091)		*B* (95%CI)	$p_{adjusted}$
		TT (n=10,392)	CT+CC (n=3,699)		
Adequacy	Having a breakfast	8.66±3.41	8.66±3.41	-.009 (-172,.154)	.946
	Mixed grains intake	2.31±2.49	2.34±2.50	-.015 (-.108,.077)	.746
	Total fruits intake	2.21±1.61	2.18±1.61	.031 (-.047,.108)	.249
	Fresh fruits intake	2.28±1.61	2.26±1.63	.020 (-.037,.076)	.494
	Total vegetables intake	3.49±1.35	3.44±1.36	.056 (-.006,.118)	.007
	Vegetables intake excluding kimchi and pickled vegetables intake	2.22±1.28	2.18±1.27	.032 (-.026,.089)	.021
	Meat, fish, eggs, and beans intake	4.63±2.53	4.55±2.53	.051 (-.053,.154)	.004
	Milk and milk products intake	4.90±4.06	4.88±4.03	-.055 (-.253,.143)	.563
	Total scores of the adequacy	30.70±9.64	30.49±9.73	.308 (.028,.588)	.031
Moderation	Ratio of white meat to red meat	2.33±2.39	2.33±2.43	-.003 (-.093,.087)	.940
	Sodium intake	6.24±3.68	6.36±3.67	-.136 (-.255,-.017)	.026
	Percentage of energy from sweets and beverages	8.04±3.62	8.18±3.50	-.131 (-.256,-.007)	.039
	Total scores of the moderation	16.61±5.65	16.87±5.57	-.264 (-.455,-.072)	.007
Energy balance	Percentage of energy from carbohydrate	2.21±1.99	2.13±1.99	.100 (.030,.170)	.005
	Percentage of energy intake from fat	3.12±2.09	3.05±2.11	.083 (.010,.156)	.025
	Energy intake	3.68±1.95	3.64±1.98	.043 (-.028,.115)	.235
	Total scores of the balance	9.01±4.56	8.81±4.57	.227 (.070,.383)	.005
Total scores of KHEI		56.32±11.98	56.17±12.07	.271 (-.110,.652)	.163

B, unstandardized coefficient; CI, confidence interval; KHEI, Korean Healthy Eating Index; N, Number of subjects. Values are expressed as means ± standard deviation. $P_{adjusted}$ values are from adjusted model controlling for covariates including age, education, cohabitation, residence, alcohol drinking, smoking, regular exercise, body mass index and total energy intake

Supplementary Table S2. KHEI scores of female study subjects by *TAS1R2* rs35874116 genotype

Domains	Items	Females (n=27,578) TT (n=20,398)	Females (n=27,578) CT+CC (n=7,180)	*B* (95%CI)	$p_{adjusted}$
Adequacy	Having a breakfast	7.77± 4.16	7.79±4.15	-.015 (-.122,.093)	.784
	Mixed grains intake	2.99±2.45	2.94±2.46	.048 (-.017,.114)	.150
	Total fruits intake	3.35±1.68	3.33±1.67	.034 (-.020,.089)	.410
	Fresh fruits intake	3.42±1.67	3.40±1.66	.017 (-.026,.060)	.433
	Total vegetables intake	3.38±1.35	3.34±1.35	.030 (-.012,.071)	.057
	Vegetables intake excluding kimchi and pickled vegetables intake	2.50±1.37	2.47±1.36	.010 (-.031,.052)	.268
	Meat, fish, eggs, and beans intake	5.03±2.72	5.02±2.70	-.012 (-.088,.064)	.849
	Milk and milk products intake	5.75±4.03	5.82±4.01	-.076 (-.208,.055)	.137
	Total scores of the adequacy	34.18±10.20	34.13±10.09	.035 (-.183,.254)	.754
Moderation	Ratio of white meat to red meat	2.98±2.90	2.99±2.91	.010 (-.068,.050)	.869
	Sodium intake	7.16±3.42	7.26±3.38	-.092 (-.172,-.013)	.023
	Percentage of energy from sweets and beverages	9.81±1.23	9.82±1.18	-.003 (-.035,.029)	.864
	Total scores of the moderation	19.95±4.47	20.06±4.41	-.102 (-.212,.009)	.071
Energy balance	Percentage of energy from carbohydrate	1.91±1.99	1.91±1.98	-.009 (-.060,.042)	.729
	Percentage of energy intake from fat	2.75±2.17	2.77±2.17	-.025 (-.079,.030)	.371
	Energy intake	3.60±2.04	3.62±2.03	-.017 (-.072,.037)	.534
	Total scores of the balance	8.26±4.32	8.30±4.32	-.052 (-.161,.058)	.359
Total scores of KHEI		62.39±11.90	62.49±11.78	-.118 (-.399,.163)	.410

B, unstandardized coefficient; CI, confidence interval; KHEI, Korean Healthy Eating Index; N, Number of subjects. Values are expressed as means ± standard deviation. $P_{adjusted}$ values are from adjusted model con-trolling for covariates including age, education, cohabitation, residence, alcohol drinking, smoking, regular exercise, body mass index and total energy intake

Supplementary Table S3. KHEI scores of obese subjects by *TAS1R2* rs35874116 genotype

Domains	Items	Obese (BMI≥25 kg/m^2) (n=13,894)		*B* (95%CI)	$p_{adjusted}$
		TT (n=10,216)	CT+CC (n=3,678)		
Adequacy	Having a breakfast	8.06±3.96	8.13±3.90	-.097 (-.278,.084)	.413
	Mixed grains intake	2.79±2.48	2.75±2.49	.037 (-.056,.129)	.436
	Total fruits intake	2.98±1.74	2.93±1.73	.066 (-.013,.145)	.166
	Fresh fruits intake	3.05±1.74	3.02±1.73	.026 (-.034,.085)	.397
	Total vegetables intake	3.44±1.34	3.40±1.38	.044 (-.016,.103)	.074
	Vegetables intake excluding kimchi and pickled vegetables intake	2.41±1.35	2.38±1.35	.025 (-.033,.083)	.177
	Meat, fish, eggs, and beans intake	4.89±2.67	4.86±2.65	.001 (-.105,.107)	.472
	Milk and milk products intake	5.39±4.06	5.38±4.05	.006 (-.182,.194)	.845
	Total scores of the adequacy	33.00±10.14	32.85±10.12	.145 (-.161,.451)	.386
Moderation	Ratio of white meat to red meat	2.79±2.80	2.73±2.71	.058 (-.046,.161)	.289
	Sodium intake	6.80±3.57	6.84±3.56	-.065 (-.179,.049)	.260
	Percentage of energy from sweets and beverages	9.19±2.50	9.24±2.43	-.047 (-.132,.038)	.053
	Total scores of the moderation	18.78±5.22	18.80±5.15	-.054 (-.226,.118)	.497
Energy balance	Percentage of energy from carbohydrate	2.00±1.98	1.99±1.98	.009 (-.062,.080)	.795
	Percentage of energy intake from fat	2.86±2.15	2.86±2.15	-.001 (-.076,.074)	.982
	Energy intake	3.53±2.05	3.48±2.07	.049 (-.026,.124)	.207
	Total scores of the balance	8.39±4.49	8.32±4.50	.057 (-.098,.212)	.473
Total scores of KHEI		60.17±12.36	59.97±12.33	.148 (-.252,.548)	.512

B, unstandardized coefficient; BMI, Body mass index; CI, confidence interval; KHEI, Korean Healthy Eating Index; N, Number of subjects. Values are expressed as means ± standard deviation. $P_{adjusted}$ values are from adjusted model con-trolling for covariates including sex, age, education, cohabitation, residence, alcohol drinking, smoking, regular exercise, body mass index and total energy intake

Supplementary Table S4. KHEI scores of non-obese subjects by *TAS1R2* rs35874116 genotype

Domains	Items	Non-Obese (BMI<25 kg/m^2) (n=27,775)		*B* (95%CI)	$p_{adjusted}$
		TT (n=20,575)	CT+CC (n=7,200)		
Adequacy	Having a breakfast	8.07±3.94	8.06±3.96	.033 (-.099,.165)	.786
	Mixed grains intake	2.75±2.48	2.73±2.49	.020 (-.046,.086)	.553
	Total fruits intake	2.96±1.74	2.95±1.74	.027 (-.030,.054)	.609
	Fresh fruits intake	3.03±1.73	3.02±1.74	.012 (-.027,.087)	.574
	Total vegetables intake	3.41±1.35	3.36±1.34	.034 (-.009,.077)	.012
	Vegetables intake excluding kimchi and pickled vegetables intake	2.39±1.35	2.37±1.33	.017 (-.025,.059)	.088
	Meat, fish, eggs, and beans intake	4.89±2.66	4.86±2.65	.021 (-.056,.099)	.187
	Milk and milk products intake	5.50±4.06	5.57±4.05	-.099 (-.235,.037)	.336
	Total scores of the adequacy	33.0±10.15	32.91±10.12	.130 (-.088,.348)	.286
Moderation	Ratio of white meat to red meat	2.74±2.73	2.78±2.80	-.028 (-.101,.045)	.423
	Sodium intake	6.87±3.52	7.01±3.48	-.126 (-.207,-.045)	.002
	Percentage of energy from sweets and beverages	9.22±2.46	9.27±2.36	-.045 (-.105,.014)	.113
	Total scores of the moderation	18.84±5.12	19.06±5.02	-.200 (-.321,-.079)	.002
Energy balance	Percentage of energy from carbohydrate	2.02±2.00	1.98±1.98	.039 (-.012,.090)	.134
	Percentage of energy intake from fat	2.88±2.15	2.87±2.15	.018 (-.036,.072)	.506
	Energy intake	3.68±1.99	3.71±1.98	-.023 (-.076,.031)	.415
	Total scores of the balance	8.58±4.37	8.56±4.36	.034 (-.076,.145)	.537
Total scores of KHEI		60.43±12.2	60.53±12.21	-.035 (-.321,.250)	.704

B, unstandardized coefficient; BMI, Body mass index; CI, confidence interval; KHEI, Korean Healthy Eating Index; N, Number of subjects. Values are expressed as means ± standard deviation. P_{adjusted} values are from adjusted model con-trolling for covariates including sex, age, education, cohabitation, residence, alcohol drinking, smoking, regular exercise, body mass index and total energy intake
